# Identification of Differentially Methylated Regions Associated with a Knockout of SUV39H1 in Prostate Cancer Cells

**DOI:** 10.3390/genes11101188

**Published:** 2020-10-13

**Authors:** Wenbo Yan, Yuqi Guo, Fangxi Xu, Deepak Saxena, Xin Li

**Affiliations:** 1 Department of Molecular Pathobiology, New York University College of Dentistry, New York, NY 10010, USA; wy12@nyu.edu (W.Y.); yg701@nyu.edu (Y.G.); fx363@nyu.edu (F.X.); ds100@nyu.edu (D.S.); 2 Perlmutter Cancer Institute, New York University Grossman School of Medicine, New York, NY 10016, USA; 3 Department of Urology, New York University Grossman School of Medicine, New York, NY 10016, USA

**Keywords:** SUV39H1, prostate cancer, methylation, cancer migration, epigenetics

## Abstract

Epigenetic alterations, such as histone methylations, affect the pathogenesis of tumors including prostate cancer (PCa). Previously, we reported that metformin reduced SUV39H1, a histone methyltransferase of H3 Lys9, to inhibit the migration of PCa cells. Since histone methylation is functionally linked to DNA methylation, we speculate that the knockout of the SUV39H1 gene will affect the genomic DNA methylation profile to regulate PCa cell migration and invasion. The genome-wide DNA methylation level is lower in SUV39H1 knockout (KO) cells than wild-type (WT) ones. However, the methylation levels in functional regions of CpG Islands (CGI), 5′ untranslated region (UTR5), and exon regions are higher in KO cells than WT cells. Analysis of differentially methylated regions (DMRs) identified 1241 DMR genes that have differential methylation on CG sites when comparing the KO and WT samples. Gene ontology enrichment and Kyoto Encyclopedia of Genes and Genomes Pathways analysis showed that knockout of SUV39H1 affects gene sets and pathways that are heavily involved in cell shapes, cell recognition, adhesion, motility, and migration. Our study suggests that SUV39H1 plays an important role in PCa migration via the epigenetic regulation of methylation on CG sites, and is a novel and legitimate target to inhibit PCa cell migration.

## 1. Introduction

Prostate cancer (PCa) is the second leading cause of cancer death in American men, behind only lung cancer. The high lethality caused by PCa is largely due to metastasis. Understanding how PCa cells metastasize and identifying new drugs to deter PCa cell motility and migration are of great clinical significance. 

According to the American Diabetes Association, 34.2 million Americans, or 10.5% of the population, had diabetes in 2018; 95% are type II diabetes. Although the mechanism of action is not fully elucidated, metformin is a widely prescribed drug for treating type II diabetes and is often the first prescribed medication. Metformin helps to manage blood glucose levels by reducing the amount of glucose produced by the liver and by increasing insulin sensitivity. Over the last 10 years, increasing evidence has demonstrated that metformin may reduce cancer risk and improve cancer prognosis and survival, including PCa [1]. Our previous work indicated that metformin inhibits salivary adenocarcinoma growth in vitro through cell cycle arrest and apoptosis [2] and targets the c-MYC oncogene to prevent PCa growth in vitro and in vivo [3]. Additionally, we reported that metformin reduced SUV39H1, a histone methyltransferase of H3 Lys9, to inhibit the migration of PCa cells via disturbing the integrin-FAK signaling [4]. This finding suggests that metformin may inhibit PCa metastasis and progression by epigenetic alterations. Chromatin is predominantly comprised of DNA, histone proteins, and RNA [5]. DNA methylation is perhaps the best-characterized epigenetic modification of chromatin [6]. Increasing evidence suggests a clear connection between covalent histone modifications such as histone methylation with longstanding epigenetic phenomena [6]. Chromatin remodeling and histone modifications, particularly the methylation of histone H3, can mediate the signaling of DNA methylation [5]. The involvement of histone methylation, particularly H3 Lys 9, plays an important role in the control of DNA methylation in multiple experimental models [5]. The interactions between DNA methylation and histone H3K9 methylation may form a reinforcing silencing loop to achieve the proper epigenetic control of the chromatin [7]. Our previous observation indicates that SUV39H1 may mediate the anti-cancer effects of metformin by inhibiting PCa cell migration, and SUV39H1 could serve as a therapeutic target for PCa treatment. We speculate that SUV39H1 is very likely to alter the status of genomic DNA methylation in PCa cells. To better understand the impact of SUV39H1 on the epigenetic regulation of cell proliferation and migration, we compared the genomic DNA methylation profile in SUV39H1 knockout (KO) and wild-type (WT) prostate cancer cells.

## 2. Materials and Methods 

### 2.1. Prostate Cell Lines and Knockout of SUV39H1

Human PCa cell line PC-3 was obtained from the American Type Culture Collection (ATCC, Manassas, VA, USA) and maintained in RPMI-1640 supplemented with 10% fetal bovine serum, 100 U/mL penicillin, and 100 μg/mL streptomycin. Cells were incubated in a 5% CO_2_ humidified incubator at 37 °C. We periodically confirmed that cells were mycoplasma-free using PlasmoTest—Mycoplasma Detection Kit (InvivoGen, San Diego, CA, USA). As described previously [4], the SUV39H1 KO cell line was created by cloning SUV39H1 sgRNA (5′-GGTTCCTCTTAGAGATACCG-3′, targeting exon 2) into Church’s vector system (Addgene, Cambridge, MA, USA, plasmid #41824) followed by co-transfection with hCas9 (Addgene plasmid #41815) and pEGFP-C1 into PC-3 cells. Cells positive for green fluorescent protein were sorted and plated into 96-well plates with single colonies expanded and screened for KO. The homozygous clones were confirmed by Western blot [4]. 

### 2.2. DNA Methylation Analysis

The whole-genome DNA was extracted in triplicates from above wild-type PC-3 cells (WT) and knockout cells (KO) and submitted to Novogene Co., Ltd. (Beijing, China) for whole-genome sequencing of bisulfite-converted DNA. Briefly, the genomic DNA samples went through fragmentation and purification, end repairing and adenylation, and adapter ligation before bisulfite conversion. Then, size selection, PCR, and library quality control were conducted before Illumina sequencing. The original data from Illumina HiSeqTMPE125/PE150 were converted to sequencing reads by base calling using CASVA and saved as FASTQ files. Clean reads were obtained by trimming using Trimmomatic software [8]; Bismark [9] was used to determine the methylation by comparing the genomic fragment and the sequence after bisulfite treatment.

### 2.3. Identification of Differentially Methylated Regions (DMRs) 

DSS analysis software (DSS_2.12.0) based on beta-binomial distribution was used for the identification of DMRs as described previously [10,11] with consideration of the spatial correlation, read depth of the sites, and the variance among biological replicates. The parameters were smoothing including span = 200, delta = 0, p.threshold = 1 × 10^−5^, minlen = 50, minCG = 3, dis.merge = 100, and pct.sig = 0.5. 

### 2.4. Gene Ontology (GO) Enrichment Analysis

GO enrichment analysis was performed according to the GOseq method [12] based on Wallenius non-central hypergeometric distribution using Goseq topGO; Bioconductor (2.13) and pathways with corrected *p*-values < 0.05 were listed. KEGG enrichment analysis was performed using KOBAS (2.0) and pathways with a corrected *p*-values < 0.05 were listed.

### 2.5. Overlapping and Annotation of Genes Altered by Methylation and Expression

We compared the methylation profile with the RNASeq data [4] from the same batch of cells. Nine hundred eighty (980) genes were showing different levels of methylation at promoter regions and different levels of expression (Supplementary Figure S1). By searching these genes in the Gene Ontology database and KEGG pathways, we annotated the related biological functions and pathways. An over-representation test was performed and a set *Q*-value (adjusted *p*-value) < 0.05 was used as a cutoff. 

## 3. Results

### 3.1. SUV39H1 Gene Function Facilitates the Maintenance of DNA-Methylation at Regulatory Elements but Not at the Gene Body

**Figure 1:**
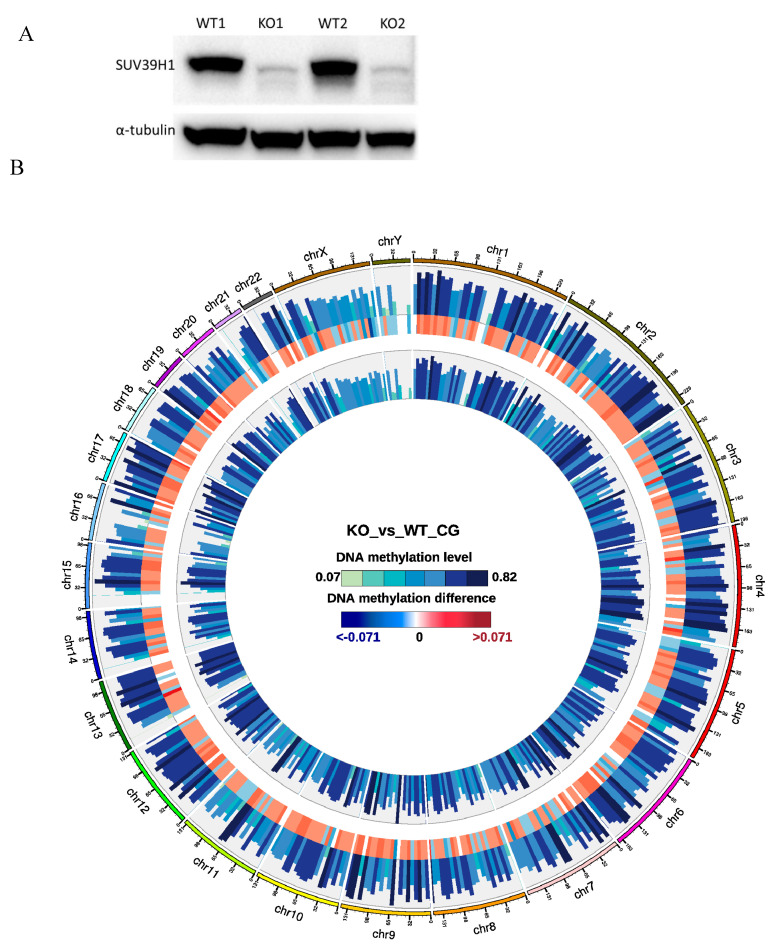
(**A**) Western blots of the total cell lysate from WT and KO cells. We extracted the proteins from total cell lysates using RIPA lysis buffer (Thermo Scientific) and determined the protein concentrations with a BCA protein assay kit (Thermo Scientific). The proteins were denatured with Bolt^®^ LDS sample buffer (Thermo Scientific) before sample loading for gel electrophoresis and membrane transfer. Primary antibodies against SUV39H1 (catalog #8729) and α-tubulin (catalog #3873, Cell Signaling Technology) were used to detect the levels of SUV39H1 and α-tubulin. The signals were detected with ECL Western Blotting substrate (Thermo Scientific) with a ChemiDoxTM XRS system (BioRad Laboratories, Inc. Hercules, CA, USA). (**B**) Circos graph comparing genome-wide methylation level. The outer circle represents the methylation level in KO samples. The inner circle represents the methylation level in WT samples. The middle circle represents the difference between methylation levels in KO and WT samples.

**Figure 2:**
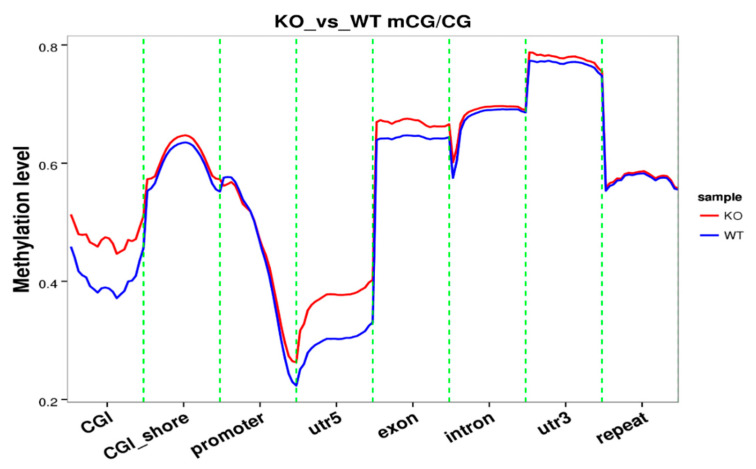
Analysis of the methylation level on functional regions in KO and WT samples.

The knockout of SUV39H1 was confirmed by Western blot (Figure 1A). A Circos graph compared genome-wide methylation levels. The methylation level is higher in some genes while lower in other genes in KO samples versus WT samples (Figure 1B). The total mC percentage is 2.47% in KO samples compared to 4.75% in the WT. In mammals, nearly all DNA methylation occurs on cytosine residues of CpG dinucleotides [6]. More specifically, the mCpG percentage is 50.49% compared to 60.94% in the WT (Table 1). Analysis of methylation level on functional regions (promoter, exon, intron, CGI, CGI shore, repeat, utr5, and utr3) involved in gene expression was performed. CpG Islands (CGI) were found in certain regulatory regions of the genome including the promoter regions of many housekeeping genes. The 5′ untranslated region (utr5) is the region of an mRNA that is directly upstream from the initiation codon. This region is important for the regulation of translation of a transcript, and is also involved in transcription regulation. The methylation level of these two regions in KO samples is significantly higher than those in WT samples (Figure 2). The methylation level of the exon region is slightly higher in KO samples than that of WT samples. The other functional regions display similar levels of methylation between the two groups.

### 3.2. Knockout of SUV39H1 Reduced the Methylation Level on the Upstream and Downstream Regions of the Gene Body When Compared to WT Cells

**Figure 3:**
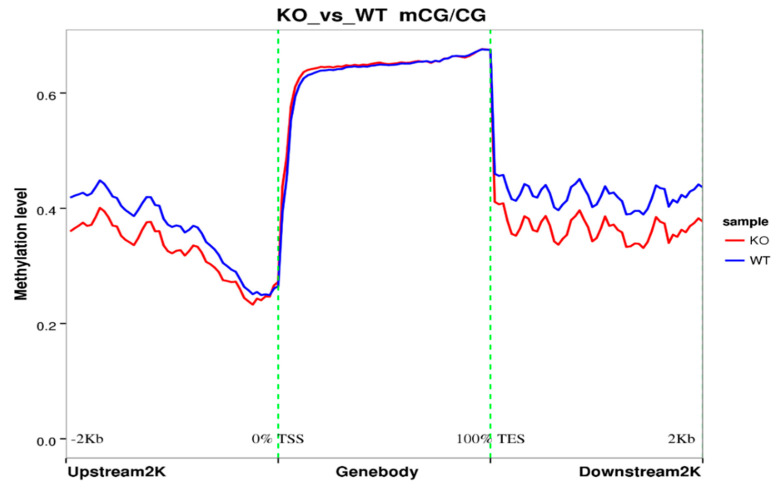
Analysis of methylation level on the upstream region (2k bp upstream from the transcription starting site) and downstream region (2k bp downstream from the transcription ending site) in KO and WT samples.

We also analyzed the upstream region (2k bp upstream from the transcription starting site) and downstream region (2k bp downstream from the transcription ending site). The methylation level of these two regions in KO samples is significantly lower than the WT samples (Figure 3). The methylation level of the gene body region is similar between the two groups (Figure 3). 

### 3.3. Identification of Differentially Methylated Regions (DMRs) and Gene Ontology (GO) Analysis

Differentially methylated regions (DMRs) are genomic regions with different methylation statuses among multiple samples. They are possible functional regions involved in gene transcriptional regulation. The identification of DMRs among multiple tissues provides a comprehensive survey of epigenetic differences among human tissues. In Table 2, the region of the Human WASH complex protein gene demonstrates differential methylation in KO and WT samples. Table 3 shows the several molecular binding functions significantly associated with the DMR-associated genes according to Gene Ontology.

**Figure 4:**
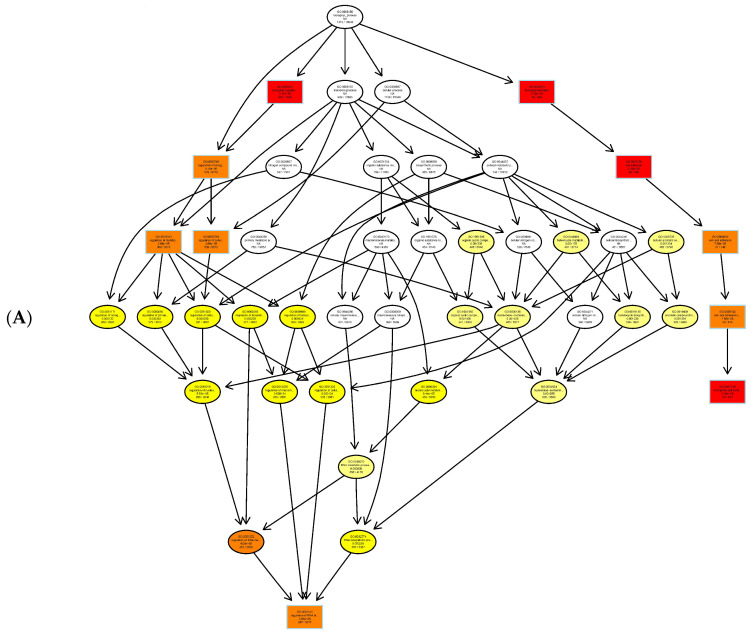

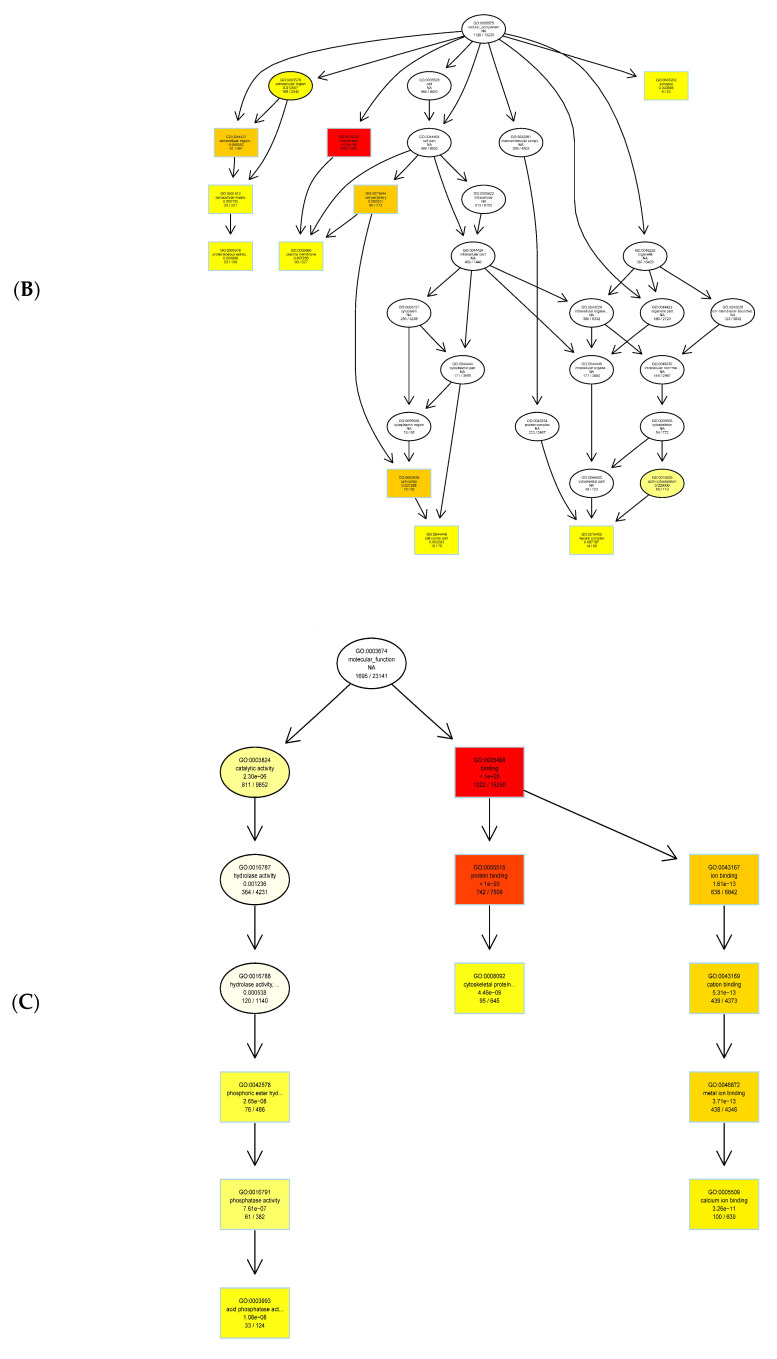

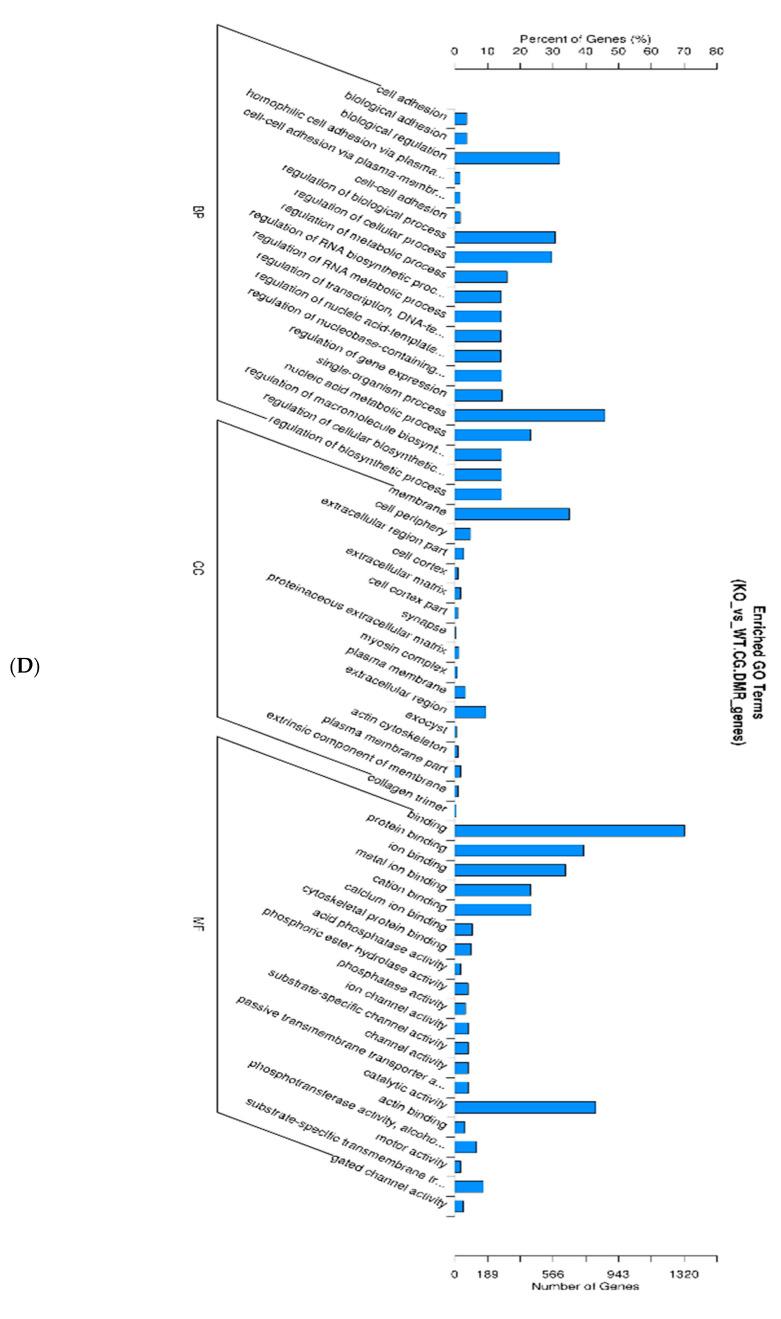
GO enrichment analysis of DMR-related genes as depicted in directed acyclic graphs (DAGs). (**A**) Biological process. (**B**) Cellular component. (**C**) Molecular functions. The top 10 enriched GO terms are enclosed in rectangles. The darker the color, the higher the degree of enrichment. *p*-Values of enrichment are shown at each node. (**D**) The summary of three independent ontologies: biological process (BP), cellular component (CC), and molecular function (MF).

The Gene Ontology resource provides a computational representation of our current scientific knowledge about the functions of genes (or, more properly, the protein and non-coding RNA molecules produced by genes). Three independent ontologies accessible on the World-Wide Web (http://www.geneontology.org) are constructed: biological process (Figure 4A), cellular component (Figure 4B), and molecular function (Figure 4C). One of the main uses of GO is to perform enrichment analysis on gene sets. For example, given a set of genes that are upregulated under certain conditions, an enrichment analysis will find the GO terms that are over-represented (or under-represented) using annotations for that gene set. GO terms are connected into nodes of a network; thus, the connections between its parents and children are known, and form what is technically described as directed acyclic graphs (DAGs). Figure 4D shows three independent ontologies.

### 3.4. KEGG Pathway Analysis in Genes with DMRs Identifies Enriched Gene Sets

**Figure 5:**
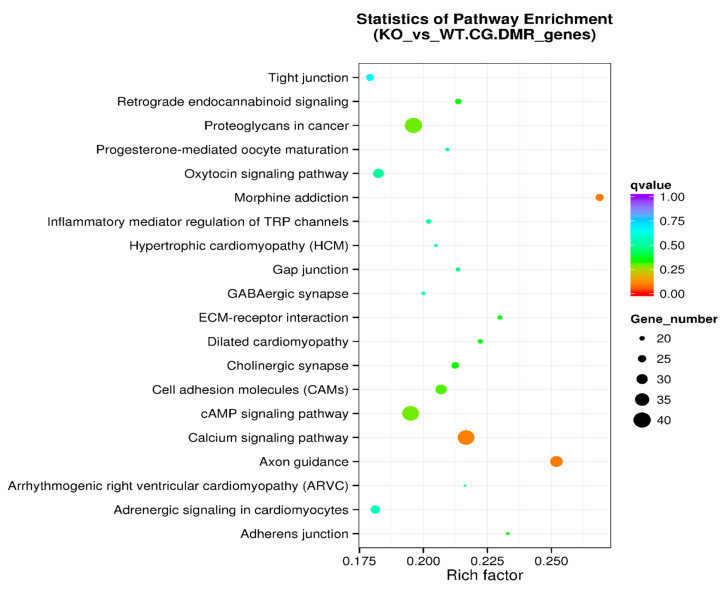
KEGG pathway enrichment analysis of DMR-associated genes in the scatter plot. The level of enrichment of the KEGG pathway is depicted as a rich factor, *Q* value, and numbers of DMR-associated genes that belong to this pathway. The rich factor is the percentage of DMR-associated genes among all the genes that belong to the pathway. The larger the rich factor, the higher the enrichment. The *Q* value (between 0 and 1) is the corrected *p*-value; the closer the *Q* value is to 0, the higher the enrichment. The size of the dot represents the number of DMR-associated genes in the pathway.

KEGG (Kyoto Encyclopedia of Genes and Genomes) is a knowledge base for the systematic analysis of gene functions, linking genomic information with higher-order functional information. The genomic information is stored in the GENES database. This is a collection of gene catalogs for all the completely sequenced genomes and some partial genomes, with up-to-date annotation of gene functions. The higher-order functional information is stored in the PATHWAY database that contains graphical representations of cellular processes such as metabolism, membrane transport, signal transduction, and cell cycle. KEGG pathway enrichment analysis of DMR-associated genes is presented in Table 4. The level of enrichment in the KEGG pathway is depicted as a rich factor, Q value, and the number of DMR-associated genes that belong to this pathway (Figure 5).

### 3.5. Gene Ontology Analysis in Gene Promoters with DMRs Identifies Enriched Gene Promoter Sets

**Figure 6:**
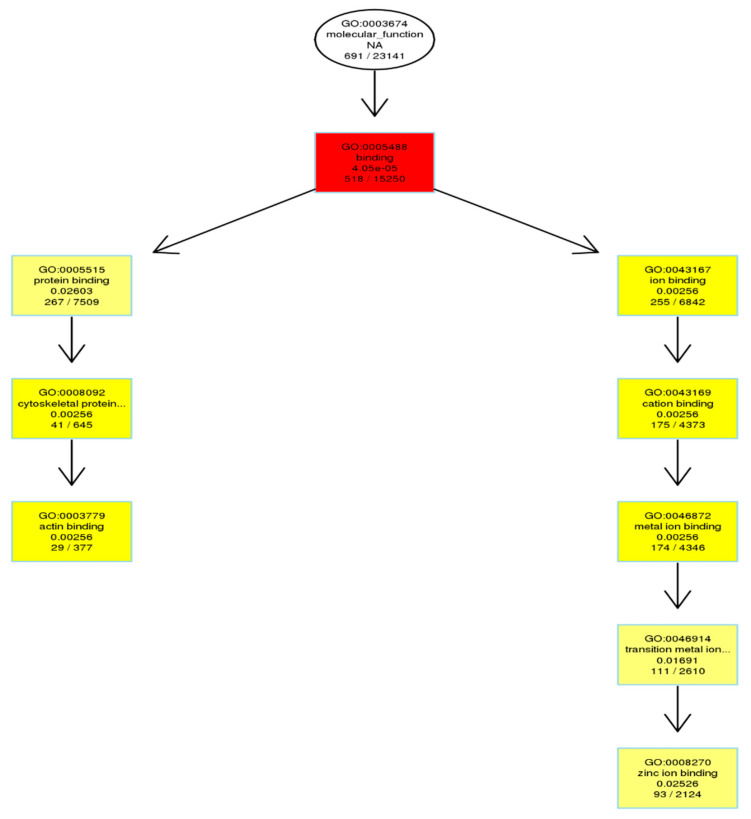
GO enrichment analysis of DMR-related gene promotors as depicted in directed acyclic graphs (DAGs). Only molecular function is shown. The top 10 enriched GO terms are enclosed in rectangles. A darker color means a higher degree of enrichment. *p*-Values of enrichment are shown at each node.

We also looked at the DMR-associated promotor regions. An enrichment analysis will find which GO terms are over-represented (or under-represented) using annotations for that gene promotor set (Table 5). As described in Result 3.3, directed acyclic graphs (DAGs) are being constructed. In Figure 6, only the DAG of the molecular function of enriched promotor sets is shown. 

### 3.6. KEGG (Kyoto Encyclopedia of Genes and Genomes) Pathway Analysis in Gene Promoters with DMRs Identifies Enriched Gene Sets

**Figure 7:**
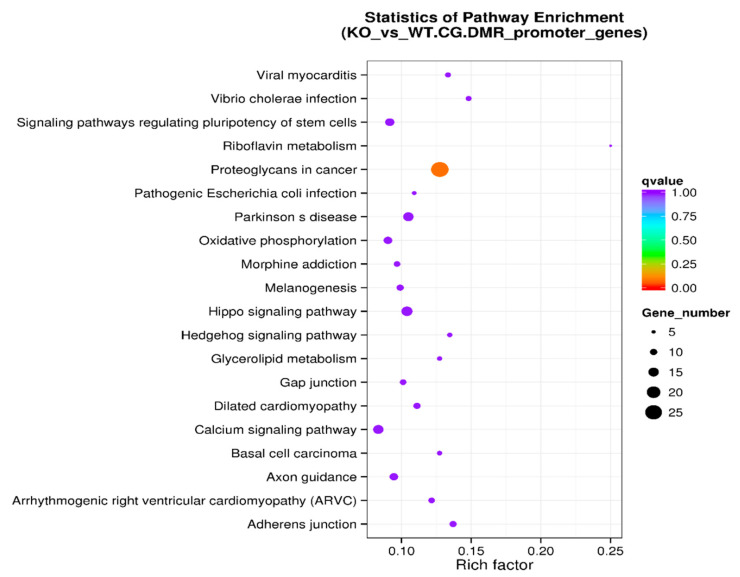
KEGG pathway enrichment analysis of DMR-associated promotors in the scatter plot. The level of enrichment of the KEGG pathway is depicted as a rich factor, *Q* value, and numbers of DMR-associated gene promotors that belong to this pathway. The rich factor is the percentage of DMR-associated gene promotors among the total gene promotors belonging to the pathway. The larger the rich factor, the higher the enrichment. The *Q* value (between 0 and 1) is the corrected *p*-value; the closer the *Q* value is to 0, the higher the enrichment. The size of the dot represents the number of DMR-associated gene promotors in the pathway.

We also performed a systematic analysis of gene functions by linking genomic information with higher-order functional information on DMR-associated promotor sets. The KEGG pathway enrichment analysis of DMR-associated promotors is presented in Table 6. The level of enrichment in the KEGG pathway is depicted in Figure 7 with the rich factor, the Q value, and the number of DMR-associated genes that belong to this pathway illustrated (Figure 7).

## 4. Discussion and Conclusions

Diverse epigenetic events collectively contribute to the regulation of chromatin structure. These events include DNA methylation, histone modification, and RNA interference [6,13]. As previously discussed in the Introduction, there is a clear functional interaction between DNA methylation and histone methylation. We speculate that the knockout of SUV39H1 might affect the methylation profile of the genomic DNA in PCa cells. Our data suggest that the knockout of the SUV39H1 enzyme significantly changed the methylation level in the KO mice genome versus the wild-type control. Although the overall methylation level in KO samples is lower than that in WT samples (Figure 1B and Table 1), the methylation level of the CpG Islands and the 5′ untranslated region in KO samples are significantly higher than those in WT samples (Figure 2). These two regions are functional regions involved in gene expression regulation. To have a comprehensive survey of epigenetic differences among KO and WT samples, differentially methylated regions (DMRs) and genomic regions with different methylation statuses were investigated. The DMRs are regarded as possible functional regions involved in gene transcriptional regulation. Table 2 shows that the DMR annotation pinpointed the target gene of the WASH complex subunit 1 (WASHC1). The WASHC1 acts as an actin nucleation-promoting factor (NPF) at the surface of endosomes, where it induces actin polymerization and plays a key role in the fission of tubules [14,15,16,17]. It regulates the trafficking of endosomal α5β1 integrin and is fundamental for integrin-mediated cancer cell invasion [18]. This is consistent with our previous observation that both metformin treatment and SUV39H1 knockout in PCa cells can reduce integrin αV and β1 proteins [4]. Moreover, endosomal WASH is also shown to facilitate the focal delivery of MT1-MMP and support pericellular matrix degradation and breast tumor cell invasion into matrix environments [19].

The Gene Ontology resource provides a computational representation of our current scientific knowledge about gene function. Three independent ontologies are being constructed: biological processes, cellular components, and molecular function. One of the main uses of GO is to perform enrichment analysis on gene sets; GO terms are gene sets over-represented (or under-represented). The GO enrichment analysis was performed on DMR-associated genes. In directed acyclic graphs (DAGs) of biological process (Figure 4A), we can see that gene sets regulating cell adhesion are enriched. In the DAG of cellular components (Figure 4B), we note that gene sets regulating the extracellular matrix, cell cortex, and actin cytoskeleton are enriched. In the DAG of molecular function (Figure 4C), gene sets regulating cytoskeleton protein-binding and calcium ion binding are enriched. Moreover, the GO enrichment analysis was performed on DMR-associated gene promotors. In the DAG of molecular function (Figure 6), we see that gene sets regulating cytoskeleton actin-binding and zinc ion binding are enriched. Cell–cell interactions are key mediators of cancer progression, and changes in cell adhesion molecules alter the ability of tumor cells to interact with other cells to facilitate immune evasion and metastatic dissemination [20,21]. Alterations in the density and composition of the extracellular matrix (ECM) contribute to tumor growth and progression [22,23]. Collagens are one of the most important components of the ECM, and have a critical function for tumor cell growth, migration, and metastasis [24].

Metastasis is a complex process requiring a dramatic reorganization of the cytoskeleton. Cytoskeletal and cytoskeleton-associated proteins have been shown to mediate tumor cell migration, invasion, and metastasis [25,26]. To undergo metastasis, cancer cells must gain the ability to migrate through the extracellular matrix and enter the bloodstream. Ca^2+^ is a crucial regulator of cell migration via the calcium signaling pathway [27]. Zinc-binding proteins could have diverse cellular roles as regulators of cytoarchitecture, cell adhesion, and cell motility [28]. More interestingly, there is a reciprocal interplay between Ca^2+^ and Zn^2+^, which regulates actin remodeling and cell migration [29]. In summary, we can see these enriched genes and gene promotor sets in KO samples are closely related to the migration, invasion, and metastasis of tumors.

The KEGG pathway analysis is a knowledge base for systematic analysis of gene functions, linking genomic information with higher-order functional information. The genomic information is stored in the GENES database, which is a collection of gene catalogs for all the completely sequenced genomes and some partial genomes with up-to-date annotation of gene functions. The higher-order functional information is stored in the PATHWAY database, which contains graphical representations of cellular processes such as metabolism, membrane transport, signal transduction, and cell cycle. KEGG pathway enrichment analysis of DMR-associated genes (Table 4 and Figure 5) identified the top five enriched pathways: axon guidance, morphine addiction, calcium signaling pathway, proteoglycans in cancer, and cAMP signaling pathway. Other enriched pathways include ECM-receptor interaction and cell adhesion molecules (Figure 5). Further analysis was performed on DMR-associated gene promotors. KEGG pathway enrichment analysis of DMR-associated gene promotors (Table 6 and Figure 7) identified the top five enriched pathways: proteoglycans in cancer, adherens junction, *Vibrio cholerae* infection, Hippo signaling pathway, and Parkinson’s disease pathway. Other enriched pathways include axon guidance, morphine addiction, and calcium signaling pathways (Figure 5).

In the normal mammary gland, axon guidance molecules are important for cell proliferation and adhesion during development. These molecules are dysregulated during breast cancer tumorigenesis and progression [30]. The role of calcium signaling pathways and cytoskeletal protein binding in cell migration and tumor metastasis was discussed in the previous section. Proteoglycans are important constituents of the extracellular matrix that help to drive multiple oncogenic pathways in tumor cells and promote critical tumor–microenvironment interactions [31]. During tumor development and growth, proteoglycan expression is markedly modified in the tumor microenvironment [32]. Downregulations of adherens junction are tightly controlled and are essential for epithelial–mesenchymal transition (EMT) during embryogenesis. The characteristics of EMT are a loss of cell adhesion and increased cellular mobility. Hence, dysregulated EMT has been associated with cancer progression and metastasis [33]. The Hippo signaling pathway regulates cell proliferation and survival. Many genes in this pathway are recognized as tumor suppressors, and the pathway’s deregulation is a key feature in many cancers [34,35]. In summary, these enriched pathways are closely related to the migration, invasion, and metastasis of tumors.

Dynamic changes in chromatin methylation are essential for cell-fate determination and development. Consequently, faulty regulation of epigenetic events including histone methylation can lead to tumorigenesis [5]. A large number of specific inhibitors of different histone methyltransferases have been developed over the past few years, as reviewed previously in [36,37]. For example, enhancer of zeste homolog 2 (EZH2), a histone methyltransferase, is linked to multiple cancers, including breast cancer, castration-resistant prostate cancer, small-cell lung cancer (SCLC), and neuroblastoma [5]. Preclinical in vivo studies have demonstrated that depletion or inhibition of EZH2 impairs tumor proliferation and growth in vivo, as reviewed by Michalak et al. [5]. Ongoing clinical trials of multiple EZH2 inhibitors demonstrated a favorable safety profile and encouraging efficacy in a range of cancers [5]. This suggests that targeting enzymes that regulate histone methylation could be a legitimate and promising therapeutic approach in the war against cancer.

In summary, our data indicate that the knockout of SUV39H1 affects gene sets and pathways that are heavily involved in cell shapes, cell recognition, adhesion, motility, and migration. Our study suggests that SUV39H1 plays an important role in PCa migration via epigenetic regulation, and is a novel and legitimate target to inhibit PCa cell migration.

## Figures and Tables

**Table 1:** Genome-wide methylation level comparison in wild-type (WT) and knockout (KO) samples. The mC percent (%) represents the percentage of methylated cytosine (C) nucleotide among all C nucleotides. The mCpG percent (%) represents the percentage of methylated cytosine (C) nucleotide in CpG among all C nucleotides in CpG. The mCHG and mCHH represent methylation of cytosine (C) nucleotide in non-CpG sites. In mCHG and mCHH, the “H” represents one of the nucleotides (A, C, or T).

Samples	mC Percent (%)	mCpG Percent (%)	mCHG Percent (%)	mCHH Percent (%)
**KO**	2.47%	50.49%	0.04%	0.04%
**WT**	4.75%	60.94%	2.02%	1.87%

**Table 2:** Annotation of differentially methylated regions (DMRs). Chr: chromosome ID; C_Number: number of C nucleotides in DMR region; AreaStat: statistical significance; C_Context: sequence context; CHH: H represents A, C, or T.

Chr	Start	End	DMR_ Length	C_ Number	Group1_ MeanMethy	Group2_ MeanMethy	Diff.Methy	AreaStat	C_ Context	RegionID	Region	Gene Name
**chr1**	29,297	30,663	1367	24	0.6739981097	0.0003592509	0.6736388588	218.210743997337	CHG	ENSG00000227232.4	intron	sp|A8K0Z3|WASH1_HUMAN WAS protein family homolog 1 OS = Homo sapiens GN = WASH1 PE = 1 SV = 2//0
**chr1**	29,297	30,663	1367	24	0.6739981097	0.0003592509	0.6736388588	218.210743997337	CHG	ENSG00000243485.2	promoter	-//-
**chr1**	29,297	30,663	1367	24	0.6739981097	0.0003592509	0.6736388588	218.210743997337	CHG	ENSG00000227232.4	exon	sp|A8K0Z3|WASH1_HUMAN WAS protein family homolog 1 OS = Homo sapiens GN = WASH1 PE = 1 SV = 2//0
**chr1**	29,297	30,663	1367	24	0.6739981097	0.0003592509	0.6736388588	218.210743997337	CHG	ENSG00000227232.4	promoter	sp|A8K0Z3|WASH1_HUMAN WAS protein family homolog 1 OS = Homo sapiens GN = WASH1 PE = 1 SV = 2//0
**chr1**	29,297	30,663	1367	24	0.6739981097	0.0003592509	0.6736388588	218.210743997337	CHG	ENSG00000243485.2	TSS	-//-

**Table 3:** Gene Ontology of DMR-associated genes. GO accession: GO ID, a unique seven-digit identifier prefixed by GO; Description: functional description. Term type: cellular_component, biological_process, or molecular_function. DMR genes item: number of DMR genes related to this GO accession. DMR genes list: GO annotation of DMR related gene number.

GO Accession	Description	Term Type	Over-Represented *p*-Value	Corrected *p*-Value	DMR Genes Item	DMR Genes List
**GO:0005488**	binding	molecular_function	1.3575 × 10^−33^	6.5321 × 10^−30^	1322	1886
**GO:0005515**	protein binding	molecular_function	6.1947 × 10^−28^	1.4904 × 10^−24^	742	1886
**GO:0043167**	ion binding	molecular_function	1.0057 × 10^−16^	1.6131 × 10^−13^	638	1886
**GO:0046872**	metal ion binding	molecular_function	3.0856 × 10^−16^	3.712 × 10^−13^	438	1886
**GO:0043169**	cation binding	molecular_function	5.5219 × 10^−16^	5.3143 × 10^−13^	439	1886

**Table 4:** KEGG pathway enrichment analysis of DMR-associated genes. Term: KEGG pathway information. ID: KEGG database ID. DMR genes number: numbers of DMR associated genes that belong to this pathway. Background number: numbers of all genes that belong to this pathway.

Term	Database	ID	DMR Genes Number	Background Number	*p*-Value	Corrected *p*-Value
**Axon guidance**	KEGG PATHWAY	hsa04360	32	127	0.000349199407078	0.0922634758409
**Morphine addiction**	KEGG PATHWAY	hsa05032	25	93	0.000675922899933	0.0922634758409
**Calcium signaling pathway**	KEGG PATHWAY	hsa04020	39	180	0.00109832960455	0.0999479940137
**Proteoglycans in cancer**	KEGG PATHWAY	hsa05205	40	204	0.00448984458428	0.293545961799
**cAMP signaling pathway**	KEGG PATHWAY	hsa04024	39	200	0.00537629966665	0.293545961799

**Table 5:** Gene Ontology of DMR-associated gene promotors. GO accession: GO ID, a unique seven-digit identifier prefixed by GO; Description: functional description. Term type: cellular_component, biological_process, or molecular_function. DMR genes item: number of DMR genes related to this GO accession. DMR genes list: GO annotation of DMR-related gene number.

GO Accession	Description	Term Type	Over-Represented *p*-Value	Corrected *p*-Value	DMR Genes Item	DMR Genes List
**GO:0005488**	binding	molecular_function	8.4069 × 10^−9^	4.0454 × 10^−5^	518	774
**GO:0003779**	actin binding	molecular_function	2.1344 × 10^−6^	0.0025633	29	774
**GO:0043167**	ion binding	molecular_function	2.2839 × 10^−6^	0.0025633	255	774
**GO:0008092**	cytoskeletal protein binding	molecular_function	2.6609 × 10^−6^	0.0025633	41	774
**GO:0043169**	cation binding	molecular_function	3.0493 × 10^−6^	0.0025633	175	774

**Table 6:** KEGG pathway enrichment analysis of DMR-associated promotors. Term: KEGG pathway information. ID: KEGG database ID. DMR genes number: numbers of DMR associated genes that belong to this pathway. Background number: numbers of all genes that belong to this pathway.

Term	Database	ID	DMR Genes Number	Background Number	*p*-Value	Corrected *p*-Value
**Proteoglycans in cancer**	KEGG PATHWAY	hsa05205	26	204	0.000308759212763	0.0778073216163
**Adherens junction**	KEGG PATHWAY	hsa04520	10	73	0.013772326611	0.989573191398
***Vibrio cholerae*** **infection**	KEGG PATHWAY	hsa05110	8	54	0.0179301014766	0.989573191398
**Hippo signaling pathway**	KEGG PATHWAY	hsa04390	16	154	0.0218962296612	0.989573191398
**Parkinson’s disease**	KEGG PATHWAY	hsa05012	15	143	0.024161195271	0.989573191398

## Supplementary Materials

The following are available online at https://www.mdpi.com/2073-4425/11/10/1188/s1. Figure S1: Heatmap of genes showing different levels of methylation at the promoter regions and different levels of expression between WT and SUV39H1_KO cells.

## Abbreviations

SUV39H1	A histone methyltransferase of H3 Lys9
PCa	Prostate cancer
KO	knockout
WT	wild-type
UTR5	5′ untranslated region
CGI	CpG Islands
mCpG	Methylation on cytosine residues of CpG dinucleotides
DMRs	Differentially methylated regions
GO	Gene Ontology
DAG	Directed acyclic graph
KEGG	Kyoto Encyclopedia of Genes and Genomes
