# Supplementary material for: Identification of Differentially Methylated Regions Associated with a Knockout of SUV39H1 in Prostate Cancer Cells
Source: Genes (Basel). 2020 Oct 13;11(10):1188. doi: 10.3390/genes11101188 (PMC7601968; doi:10.3390/genes11101188)
Supplement: Supplementary file 1 [file genes-11-01188-s001.pdf]

Supplementary Figure 1.

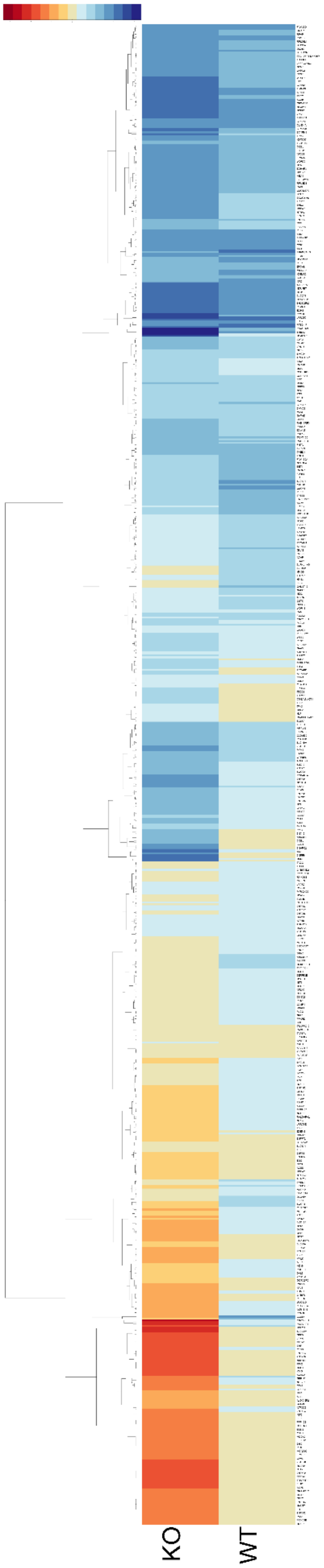

Legend:

Heatmap of genes showing different levels of methylation at the promoter regions and different levels of expression between WT and SUV39H1_KO cells. The methylation profile was compared with the RNASeq data from the WT and SUV39H1_KO cells and 980 genes were showing differences both in the levels of methylation at the promoter regions and the levels of expression between WT and KO cells.
